# Endocrine changes in SARS-CoV-2 patients and lessons from SARS-CoV

**DOI:** 10.1136/postgradmedj-2020-137934

**Published:** 2020-06-11

**Authors:** Shubham Agarwal, Sanjeev Kumar Agarwal

**Affiliations:** Department of Internal Medicine, Rosalind Franklin University of Medicine and Science Chicago Medical School, North Chicago, Illinois, USA; Department of Cardiology, Rashid Hospital, Dubai, United Arab Emirates

**Keywords:** general endocrinology, thyroid disease, adrenal disorders, general diabetes

## Abstract

Coronavirus infection outbreaks have occurred frequently in the last two decades and have led to significant mortality. Despite the focus on reducing mortality by preventing the spread of the virus, patients have died due to several other complications of the illness. The understanding of pathological mechanisms and their implications is continuously evolving. A number of symptoms occur in these patients due to the involvement of various endocrine glands. These clinical presentations went largely unnoticed during the first outbreak of severe acute respiratory syndrome (SARS) in 2002–2003. A few of these derangements continued during the convalescence phase and sometimes occurred after recovery. Similar pathological and biochemical changes are being reported with the novel coronavirus disease outbreak in 2020. In this review, we focus on these endocrine changes that have been reported in both SARS coronavirus and SARS coronavirus-2. As we battle the pandemic, it becomes imperative to address these underlying endocrine disturbances that are contributing towards or predicting mortality of these patients.

## Introduction

On 31 December 2019, the WHO office in China was informed of a few cases of pneumonia of unknown cause originating from the city of Wuhan, Hubei in China.^1^ Over the next 3 months, the severe acute respiratory syndrome coronavirus-2 (SARS-CoV-2) illness commonly called COVID-19 has affected more than four million people globally with over 1.4 million cases in the USA itself, making it the new epicentre of the illness followed by much of Western Europe. The mortality rates from different parts of the world show vast differences but studies have consistently shown that the presence of comorbidities, specially type 2 diabetes mellitus significantly increases mortality in patients with SARS-CoV-2 with an OR of 2.85.^2–5^ A study from China also showed that patients in the intensive care unit with SARS-CoV-2 infection were more likely to have underlying diabetes (22.2% vs 5.9%) than others.^6^ Certain forms of hypophysitis, thyroiditis and adrenalitis have viral aetiologies. Changes in endocrine functions observed during the previous coronavirus outbreak with SARS-CoV, led to considerable morbidity and were important predictors of mortality. Therefore, it is imperative to know the endocrinopathic impact of SARS-CoV-2 illness. The purpose of this review is to highlight the available data on endocrine changes in such patients and to recapitulate the existing knowledge from previous coronavirus outbreaks for better risk stratification and management of patients with SARS-CoV-2 infection.

## Methods

**Figure 1:**
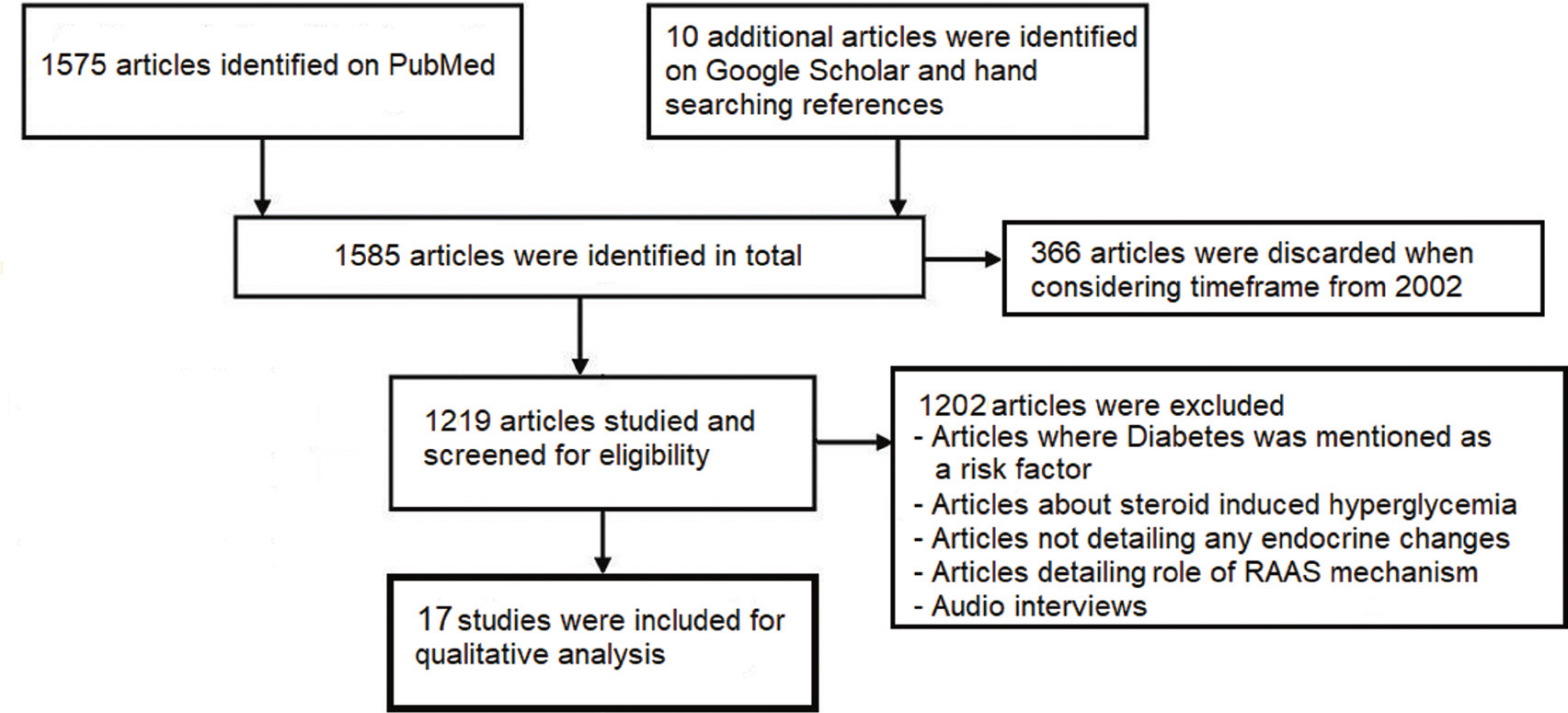
Flow chart of literature search and selection of studies. RAAS, renin-angiotensin aldosterone system.

All articles reporting endocrine changes in SARS-CoV-2 and SARS-CoV patients were considered. Electronic database of PubMed was searched using medical subject headings (MeSH) terms related to SARS-CoV-2, SARS-CoV and different hormones. Since the SARS-CoV outbreak occurred in 2002, search results were limited to January 2002 onwards to consider articles pertaining to the outbreaks of both the viruses. A considerable number of articles were in the Chinese language and were included after being translated into English. This was supplemented by handsearching reference lists for additional studies and case reports. Similarly, Google Scholar database was handsearched. Studies listing type 2 diabetes mellitus as a risk factor of SARS-CoV-2 and SARS-CoV were excluded since they did not mention any biochemical changes. Articles about corticosteroid induced hyperglycaemia, audio interviews and articles detailing the renin-angiotensin aldosterone system (RAAS) inhibitors were excluded. An initial search showed 1585 articles; finally, 17 of them were included (figure 1).

## Discussion

**Figure 2:**
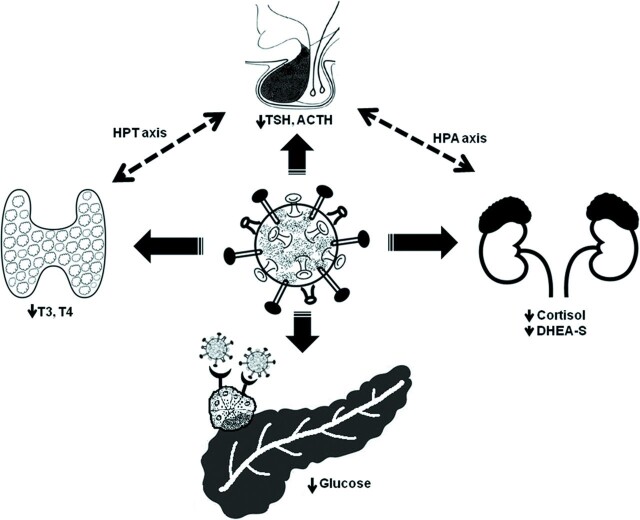
Schematic representing endocrine changes seen in SARS-CoV infections. ACTH, adrenocorticotropic hormone; DHEA-S, dehydroepiandrosterone sulfate; HPA, hypothalamic–pituitary–adrenal; HPT, hypothalamo–pituitary–thyroid; SARS-CoV, severe acute respiratory syndrome coronavirus; TSH, thyroid stimulating hormone.

Coronavirus infections cause a systemic disease that injures many organs. These patients manifest many hormonal and metabolic disturbances. Changes in thyroid, pancreas and adrenal glands have been reported in detail in both SARS-CoV and the current SARS-CoV-2 infections. The pathology data, clinical features, management and outcome of these endocrinopathies are highlighted below (figure 2).

### Thyroid changes

A detailed histopathological study on the effects of SARS infection on the thyroid gland was done during the SARS-CoV outbreak.^7^ There was extensive injury of the parafollicular cells and follicular epithelium with the destruction and exfoliation of epithelial cells into the follicle leading to its disruption. These changes were responsible for lower levels of thyroid hormones noted in clinical studies. Parafollicular cells are responsible for calcitonin production and they could not be identified on H&E sections in the tissue sample from patients with SARS. Damage to interfollicular tissue including parafollicular cells may adversely affect calcitonin production. An interesting finding was the absence of inflammatory infiltrate and features of cellular necrosis, supporting the hypothesis of extensive apoptosis responsible for thyroid injury.

The pathology data of the thyroid gland in patients with SARS-CoV-2 infection have been published by Yao *et al*. They conducted minimally invasive autopsies from different organs in patients who had died of SARS-CoV-2 and investigated the pathological changes.^8^ They reported no abnormalities in the thyroid follicular morphology but did note lymphocytic infiltration in the interstitium. None of the tissue immunohistochemistry and PCR analyses detected SARS-CoV-2 in the thyroid gland. These findings are at variance with those reported in SARS-CoV though the patient characteristics in the two studies were nearly the same. However, irrespective of pathological findings, most of the studies found a similar pattern of biochemical changes.

Limited data suggest that the thyroid gland and the hypothalamo–pituitary–thyroid (HPT) axis are directly affected in SARS-CoV-2 illness. A study of 274 patients with SARS-CoV-2 showed that thyroid stimulating hormone (TSH) and free triiodothyronine (T3) concentrations were significantly lower in deceased patients (0.7 mIU/mL and 2.8 pmol/L) than in recovered patients (1.4 mIU/mL and 4.3 pmol/L). The difference in the thyroxine (T4) levels was not statistically significant.^9^

A study by Wang *et al* reported that serum levels of T3 and T4 in patients with SARS-CoV were significantly lower than those in the control group, and low T3 levels correlated with disease severity. Serum T3 and T4 levels were decreased, respectively, in 94% and 46% of 48 patients with SARS-CoV during the acute phase and 90% and 38% during the convalescent phase of the disease.^10^ The authors also noted lower levels of TSH in these patients. Dysfunction of the HPT axis caused a diminished level of serum TSH in patients with SARS-CoV despite low serum T3 and T4. No significant difference in serum parathyroid hormone level was observed between patients with SARS-CoV and the control group, during the acute or convalescent phases of the illness.

Changes in thyroid hormone levels may persist after clinical recovery. Leow *et al* studied SARS-CoV survivors for hormonal derangements 3 months after recovery and noted that 6.7% of the patients had developed biochemical hypothyroidism.^11^ The majority of these patients (75%) had a central aetiology and only 25% developed primary hypothyroidism with positive antibodies and continued to receive thyroid hormone replacement at the end of the study. On follow-up, changes in the thyroid profile of the patients with central hypothyroidism had normalised. Central hypocortisolism was also present in 66% of patients who had central hypothyroidism. The authors suggested a dysfunctional HPT axis as evidenced by the results of thyroid hormones and the coexistence of central hypocortisolism.

These studies suggest for the monitoring of thyroid function tests during acute illness as well as during convalescence of SARS-CoV-2 with the possibility of replacement therapy as indicated. There were reports of recovery of thyroid function in 3– 6 months in SARS-CoV patients, implying reassessment of survivors of the current pandemic.^11^

### Endocrine pancreas changes and diabetes

Many viruses, such as enteroviruses, Coxsackie B virus,^12^ retroviruses, rubella, mumps, cytomegalovirus, Epstein-Barr and varicella zoster virus have been implicated in the development of diabetes mellitus in humans.^13^

Coronavirus has also been reported to cause diabetes in animal and human studies. In a case report of a foal, it was suggested that the pancreas could have had direct viral damage due to coronavirus leading to diarrhoea and transient diabetes mellitus simultaneously.^14^

It has been demonstrated that coronaviruses enter human lungs, pancreas and other tissues by interacting with the ACE2 receptor. The expression of ACE2 is much stronger in pancreatic endocrine tissue compared with the exocrine tissue.^15^ The extent of tissue damage by SARS-CoV is a direct function of the level of expression of tissue ACE2. Thus, SARS-CoV viruses may damage pancreatic islets and cause acute insulin dependent diabetes mellitus but not acute pancreatitis.

The pancreatic biopsy findings in SARS-CoV-2 patients have been identical to the previous studies in SARS-CoV patients. Yao *et al* reported no obvious abnormalities in the epithelial cells in the exocrine pancreas; however, degeneration of a few islet cells was noted.^8^

It was observed that patients with mild SARS-CoV who did not receive glucocorticoid medications during the disease course had a higher level of fasting plasma glucose (FPG) on the first day of hospitalisation than those who had non-SARS pneumonia. New-onset diabetes developed in 51% of SARS-CoV patients who had no previous diabetes and received no steroid treatment during illness.^15^ Hyperglycaemia was an independent predictor for mortality and morbidity in SARS-CoV patients with and without a history of diabetes mellitus.^4^ Yang *et al* demonstrated that before steroid treatment, the mean FPG level was significantly higher in SARS-CoV patients (deceased vs survivors vs non-SARS pneumonia group: 9.7±5.2 vs 6.5±3.0 vs 5.1±1.0 mmol/L, p<0.01). Hyperglycaemia (FPG≥7.0 mmol/L) before steroid treatment was significantly associated with death (OR 3.3) after adjustment for age and gender. There was an incremental rise in mortality with increasing FPG. Another interesting association noted was between hypoxaemia and higher FPG levels.^4^ Among patients with no known history of diabetes and before the commencement of steroid therapy, those who had hypoxaemia (arterial oxygen saturation <93%) had higher FPG levels than those who did not have hypoxia in both the survivor (8.7±4.9 vs 6.3±2.1 mmol/L) and deceased (9.8±4.8 vs 7.2±1.5 mmol/L) groups. It was estimated that for every 1.0 mmol/L increase in FPG, the HR increased by 1.08-fold for death and 1.07-fold for hypoxia morbidity. In both survivors and deceased patients, irrespective of steroid dosage, elevated FPG values started falling in the third week indicating gradual recovery of islet cell function.

About half a billion people are currently living with diabetes worldwide. Patients with diabetes had a poorer outcome in previous epidemics of SARS-CoV and H1N1 epidemics.^16  17^ The clinical course of SARS-CoV-2 infection in patients with diabetes and without other comorbidities was recently reported by Guo *et al* from Wuhan, China.^18^ Patients with diabetes may present with milder symptoms initially, but they were at higher risk of rapid progression to severe pneumonia, uncontrolled cytokine storm and hypercoagulable state contributing to a poorer prognosis. Interleukin-6 (IL-6) level was found to be higher in SARS-CoV-2 cases with diabetes than those without diabetes. Wang *et al* hypothesised that SARS-CoV-2 infection in patients with diabetes might trigger stress condition and increase secretion of hyperglycaemic hormones, such as glucocorticoid and catecholamines, resulting in elevated blood glucose, abnormal glucose variability and diabetic complications in turn leading to higher mortality and morbidity rates in these patients.^19^

A study of 99 SARS-CoV-2 patients demonstrated that 52% of patients had elevated blood glucose with a mean glucose value of 7.4 mmol/L.^20^ Wu *et al*^21^ showed that SARS-CoV-2 patients developing acute respiratory distress syndrome (ARDS) had higher glucose levels (7.4 vs 5.4 mmol/L) compared with patients who did not develop ARDS (HR 1.13). Similarly, in SARS-CoV-2 patients presenting with gastrointestinal symptoms, increased glucose level was an independent risk factor for severe/critical illness on multivariate analysis (OR 2.42).^22^ SARS-CoV-2 patients with diabetes are at high risk of progressing rapidly with ARDS and septic shock. Ma *et al* recommended FPG of 4.4–6.1 mmol/L, 2-hour postprandial plasma glucose (2 h PG) 6.1–7.8 mmol/L in mild cases and FPG 7.8–10.0 mmol/L, 2 h PG 7.8–13.9 mmol/L for critically ill patients of SARS-CoV-2.^23^

To sum up, SARS-CoV infections damage islet cells causing acute temporary diabetes. High FPG and associated metabolic changes lead to increased morbidity and mortality in these patients. In addition, pre-existing diabetes may be considered a risk factor for poorer outcomes of SARS-CoV-2 pneumonia. Intensive blood glucose monitoring and insulin therapy to obtain optimal metabolic control may improve the outcome of these patients. Tocilizumab, a monoclonal antibody against IL-6 may have a role in patients with diabetes to control overexpressed IL-6. It is being used off-label currently and a randomised controlled trial is testing its utility.^24^ The European Society of endocrinology statement advises patients with diabetes to avoid physician office visits and seek telephonic/electronic consultations for optimal control of blood sugar. They need to get an early referral to emergency services in case of possible symptoms of SARS-CoV-2.^25^

### Adrenal gland changes

The histological changes in adrenal glands of the patients dying from SARS-CoV were described by Ding *et al* from postmortem biopsy samples.^26^ They noted that the adrenal medulla had been infiltrated by mainly the monocytes and lymphocytes. Another interesting finding was the focal necrosis of the gland. In addition, vasculitis of the small veins of the adrenal medulla was present. No similar studies have been conducted in SARS-CoV-2 patients until date. However, there are reasons to believe that similar adrenal gland changes could occur given the similarity of the viral structure and mechanism of entry into human host cells.

SARS-CoV genome sequences have been detected in the cytoplasm of numerous neurons in the hypothalamus and cortex.^27^ It might induce either hypophysitis or directly affect the hypothalamus leading to hypothalamic–pituitary dysfunction. Leow *et al*^11^ studied hypocortisolism in patients who had recovered from SARS-CoV. Patients with an intact hypothalamic–pituitary–adrenal (HPA) axis at the time of acquiring SARS-CoV were included in the study. These patients also had symptoms like lethargy, malaise, lassitude, fatigue, weakness, orthostatic dizziness, anorexia, apathy, anxiety and depression after recovery. Three months after recovery, 39·3% of patients had hypocortisolism, of which 83·3% had central hypocortisolism as depicted by low adrenocorticotropic hormone (ACTH) levels. It was interesting to note that the majority of these patients had not received any systemic glucocorticoids as part of the treatment for SARS ruling out the possibility of HPA axis suppression by exogenous corticosteroid use. The hypocortisolism was, however, transient and resolved in 62·5% of patients within a year with an average duration of 5·9±3·1 months. These patients also reported resolution of symptoms including orthostatic hypotension in comparison to the start of the study. Six patients (25%) continued with residual hypocortisolism at 1-year follow-up and one of them still required hydrocortisone replacement. A quarter of the patients also revealed transient biochemical thyroid changes with some patients developing both a central hypothyroid and hypoadrenal state with the HPA axis affected more frequently than the HPT axis. The authors also found low levels of dehydroepiandrosterone sulphate (DHEAS) in 13.1% patients after adjusting for age, sex and menopausal status. DHEAS replacement could further ameliorate symptoms in those with DHEAS deficiency.

Findings from SARS-CoV and SARS-CoV-2 patients suggest for actively monitoring biochemical changes like serum cortisol and ACTH. Shall these biochemical changes go unnoticed; it may complicate post-SARS recovery. Symptomatic patients with orthostatic hypotension may require physiological doses of hydrocortisone replacement until HPA axis recovery. In addition, patients with adrenal insufficiency and Cushing’s syndrome are at increased risk of SARS-CoV-2 infection and should adhere to enhanced preventive steps.

### Miscellaneous

Changes in the cells of anterior pituitary were noted in biopsy samples of SARS-CoV patients.^28^ On immunohistochemical staining, the number of somatotrophs, thyrotrophs and corticotrophs was reduced while the number of lactotrophs and gonadotrophs was significantly increased. These findings correlated with biochemical abnormalities of increased prolactin, luteinising hormone and follicle stimulating hormone in SARS-CoV reported earlier in Chinese literature.

Seasonal and the H1N1 influenza A virus (H1N1) pandemic have been associated with increased severity and greater risk of morbidity and mortality in obese persons compared with normal weight persons. Being overweight and obesity, both negatively impact cellular immune response to H1N1 influenza.^29^ Obesity is associated with several chronic diseases like diabetes mellitus, obstructive sleep apnoea and surfactant dysfunction in the lungs which may increase the risk of complications of SARS-CoV-2. The intensive care management of patients with severe obesity is always very challenging and difficult.^30^ It is likely that obesity is an independent risk factor for severe SARS-CoV-2 illness and should be further investigated.

## Conclusion

Early studies have linked the increased mortality rates in SARS-CoV-2 patients with the presence of diabetes mellitus. Increased glucose levels have been associated with the development of ARDS and worsening of SARS-CoV-2 illness requiring intubation and mechanical ventilation. Hyperglycaemia can be a useful predictor to assess patients and triage them effectively to provide early management strategies. Strict glucose management is required in such patients to reduce mortality. Central hypothyroidism and central hypocortisolism seem to occur commonly in patients infected with coronavirus. These changes are usually transient and occur during the illness or after recovery. This crucial information calls for reassessment of survivors of the current pandemic, and identifying these changes early with prompt management can prevent further complications. Prospective randomised clinical trials, cohort studies and pathology studies of SARS-CoV-2 patients are ongoing and may further elucidate the mechanisms of these endocrinopathies translating into better treatment of patients affected by this virus.

**Main messages**

- Endocrine abnormalities occur commonly in coronavirus infections
- Pre-existing type two diabetes mellitus increases mortality in COVID-19.
- Hyperglycaemia/new onset diabetes is an important predictor of worse outcomes in COVID-19.
- Central hypothyroidism and hypoadrenalism occur due to the disruption of the hypothalamo-pituitary-thyroid and hypothalamo-pituitary-adrenal axis.

**Current research question**

- Will intensive monitoring and a strict control of blood sugar improve morbidity and mortality in patients of COVID-19?
- Adrenal gland changes in patients of COVID-19.
- What are the chronic endocrine sequelae in SARS survivors.

**Key references**

- Yang JK, Feng Y, Yuan MY, et al. Plasma glucose levels and diabetes are independent predictors for mortality and morbidity in patients with SARS. *Diabet Med*. 2006;23:623–8.
- Wei L, Sun S, Xu CH, et al. Pathology of the thyroid in severe acute respiratory syndrome. *Human Pathology*. 2007;38(1):95–2.
- Leow MK, Kwek DS, Ng AW, et al. Hypocortisolism in survivors of severe acute respiratory syndrome (SARS). Clin Endocrinol (Oxf). 2005;63:197–2. doi:10.1111/j.1365-2265.2005.02325.x
- Yang J, Lin S, Ji X, Guo L. Binding of SARS coronavirus to its receptor damages islets and causes acute diabetes. *Acta Diabetol*. 2010;47:193–9.
- Ma WX, Ran XW. The management of blood glucose should be emphasized in the treatment of COVID-19. *J Sichuan Uni* (Medical science edition). 2020;51(2):146–0. doi:10.12182/20200360606.

**Self assessment questions**

- Which of the following viruses are diabetogenic?
  - Coxsackie B virus
  - Cytomegalovirus
  - Retroviruses
  - Coronaviruses
  - All of above
- Following changes in thyroid function test may occur in patients with SARS-CoV infection
  - Low TSH level
  - Low T3 level
  - Low T4 level
  - All of above
- Hyperglycaemia is an important predictor of morbidity and mortality in COVID-19 patients
  - True
  - False
- Monitoring of following adrenal hormones is suggested in patients with SARS illness
  - Serum cortisol
  - ACTH
  - DHEAS
  - All of above
- SARS-CoV mainly causes primary hypocortisolism
  - True
  - False

**Answers**

- E
- D
- A
- D
- B
